# Assessment of Drug Prescription Pattern in Mekelle General Hospital, Mekelle, Ethiopia, Using World Health Organization Prescribing Indicators

**DOI:** 10.1155/2020/3809157

**Published:** 2020-06-27

**Authors:** Zewdu Yilma, Mesfin Liben

**Affiliations:** ^1^Department of Pharmacy, College of Medicine and Health Science, Bahir Dar University, Bahir Dar, Ethiopia; ^2^School of Pharmacy, College of Health Science, Mekelle University, Mekelle, Ethiopia

## Abstract

**Introduction:**

Irrational prescribing is a major cause for irrational drug use. Bad prescribing habits lead to ineffective and unsafe treatment, exacerbation or prolongation of illness, distress and harm to the patient, and higher costs. Incidence of irrational prescribing practice cannot be reduced without a critical intervention by assessing the causes.

**Objectives:**

The objective of this study was to assess drug prescription pattern, using WHO prescribing indicators, in OPD at Mekelle General Hospital (MGH) *Methodology*. The study was conducted at Mekelle General Hospital (MGH), Mekelle, Northern Ethiopia, from December 2016 to April 2017. A descriptive cross-sectional, retrospective hospital-based study design was used to assess prescriptions at OPD in MGH from 01 January to 31 December 2016. A systematic random sampling technique was used to select prescriptions at the time of data collection.

**Result:**

384 prescriptions were analyzed. 751 medications were prescribed from which 679 (90.4%) were with their generic name, 225 (58.6%) prescriptions contained antibiotics, 162 (42.2%) prescriptions were encountered with injection, and 648 (86.3%) encountered from the hospital list of medicine.

**Conclusion:**

In general, average number of drugs per encounter, generic prescribing, and the use of EDL/formulary of the hospital to prescribe drugs reviewed in this study were totally out of the recommended values and hence inappropriate. The study also revealed overprescribing of both antibiotics and injections.

## 1. Introduction

Globally, more than 50% of all medicines are prescribed, dispensed, or sold inappropriately, and half of the patients fail to take them as required. In addition, about 33% of the world's population is unable to access essential medicines. The use of too many medicines per patient (polypharmacy), inappropriate use of antimicrobials for nonbacterial infections, overuse of injections when oral formulations would be more appropriate, and failure to prescribe in accordance with clinical guidelines are a common trend for irrational use of drugs [1, 2]. So, to maximize benefits and to promote human wellbeing, drugs have to be used rationally [3]. Rational drug use is the means by which drugs would be taken for their appropriate clinical needs, in doses that meet patients' own individual requirements for an adequate period of time, at the lowest cost to them and their community [4].

Prescriptions are one of the prescribing standards to promote the rational use of drugs. It is a written therapeutic transaction between the prescriber and dispenser. It is a written order by the prescriber to the dispenser on how the medicine should be dispensed. It serves as a means of communication among the prescriber, dispenser, and medicine consumer pertaining to treatment or prophylaxes [1].

Irrational prescribing is a major cause for irrational drug use. Bad prescribing habits lead to ineffective and unsafe treatment, exacerbation or prolongation of illness, distress and harm to the patient, and higher costs [4].

Researches done in some Ethiopian public Hospitals showed that all the prescribing indicators were out of the ranges recommended by World Health Organization (WHO) implying that there is a deep-rooted irrational prescribing practice in the country [5–7].

Incidence of irrational prescribing practice cannot be reduced without a critical intervention by assessing the causes. The Ethiopian government and different nongovernmental organizations (NGOs) have been doing great things to improve the rational prescription pattern by conducting different capacity building trainings such as Integrated Pharmaceutical Logistics System (IPLS) to health professionals. But, assessment of drug use pattern has not been regularly conducted in most of the government health facilities, like in Mekelle General Hospital (MGH), in which many patients are being served, and this has its own limits on the intervention to promote rational use of drugs. So, this study was attempting to assess the prescribing pattern in the outpatient department (OPD) at MGH using WHO prescribing indicators.

## 2. Method

### 2.1. Study Setting

The study was conducted at Mekelle General Hospital (MGH), Mekelle, Northern Ethiopia. Mekelle is the capital city of Tigray Administrative Regional State and it is located 783 km far from Addis Ababa. The Hospital was established in 1962. Currently, it is serving more than 500,000 people from different regions.

### 2.2. Study Design

We followed the methods of Desalegn [4] with some modification with regard to exclusion criteria.

Descriptive cross-sectional, retrospective hospital-based study design was used to assess prescriptions. The prescriptions were selected by using a systematic random sampling method until the desired sample size was achieved. Prescriptions were assessed according to the WHO and International Network for Rational Use of Drugs (INRUD) guideline.

### 2.3. Data Collection and Analysis

Three well-trained pharmacy personnel collected data on prescribing indicators retrospectively by using structured data collection forms. For this particular study, 384 prescriptions were collected retrospectively from more than 10,000 prescriptions written for a 1-year period from January 2016 to December 2016. This indicator study excluded all prescriptions that contain only antituberculosis and antiretroviral medications, medical supplies, topical preparations, nutritional supplements, and prescriptions for inpatients. The sample was selected using a systematic random sampling method, and the sampling unit was patient encounters taking place at the outpatient health facility for those patients who were ambulatory of any age. All data in the prescription collection form were first analyzed manually using Microsoft Excel 2007. In the statistical analysis, frequencies, averages/means, standard deviations, and percentages were obtained.

### 2.4. Prescribing Indicators

The WHO prescribing indicators were used in this study [8]. The indicators were pretested, and a slight modification was made so that they could be used easily to provide accurate data. The final versions of the pretested indicators are described below.

The prescribing indicators that were measured included the following:

- The average number of drugs prescribed per encounter was calculated to measure the degree of polypharmacy. It was calculated by dividing the total number of different drug products prescribed by the number of encounters surveyed. Combinations of drugs prescribed for one health problem were counted as one
- Percentage of drugs prescribed by generic name was calculated to measure the tendency of prescribing by generic name. It was calculated by dividing the number of drugs prescribed by generic name by the total number of drugs prescribed, multiplied by 100
- Percentage of encounters in which an antibiotic prescribed was calculated to measure the overall use of commonly overused forms of drug therapy. It was calculated by dividing the number of patient encounters in which an antibiotic was prescribed by the total number of encounters surveyed, multiplied by 100
- Percentage of encounters with an injection prescribed was calculated to measure the overall level use of commonly overused forms of drug therapy. It was calculated by dividing the number of patient encounters in which an injection was prescribed by the total number of encounters surveyed, multiplied by 100
- Percentage of drugs prescribed from an Essential Medicines List (EML) was calculated to measure the degree to which practices conform to a national drug policy as indicated in the essential drug list of MGH. Percentage is calculated by dividing the number of products prescribed which are in the essential drug list by the total number of drugs prescribed, multiplied by 100

### 2.5. Operational Definition

#### 2.5.1. Generic Name

The generic name is an international nonproprietary name of drugs described in the Essential Medicines List (EML) 4^th^ Edition 2010, Essential Medicines Formulary 2^nd^ Edition 2013, or Standard Treatment Guideline 3^rd^ Edition, 2014.

#### 2.5.2. Essential Drug List

The essential drug list is a document which contains essential drugs, which is the Ethiopian EML4^th^ Edition, 2010.

#### 2.5.3. Combination of Drugs

Combination of drugs is described as two or more drugs which are prescribed for a single health problem for a patient.

#### 2.5.4. Polypharmacy

Polypharmacy is prescribing two or more drugs in a single prescription for a single patient.

### 2.6. Ethical Considerations

The research proposal was approved by the Ethical Clearance Committee of Mekelle University College of Health Science, School of Pharmacy. Written permission of the college was secured for the study, and the head (CEO/Medical Director) of MGH was informed about the objectives of the study and had consent from the Hospital. Confidentiality was ensured by making the data collection format to not include any patient/individual information (like name, address).

## 3. Results

384 prescriptions were analyzed, and a total of 751 prescribed drug products were obtained. The average number of drugs per prescription was 1.96. The total number of drugs prescribed by generic name was 679 (90.4%). Antibiotics were prescribed in 225 (58.6%) encounters and injections were prescribed in 162 (42.2%) encounters. 648 (86.3%) drugs prescribed were from the essential drug list of MGH (Table 1).

231 (60%) of the prescriptions contained two or more drugs and 153 (39.8%) of the encounters had contained only one drug product.

Antibiotics were the most commonly prescribed drug classes (225 (58.6%)) of encounters followed by analgesics and antipyretics (164 (42.7%)) and antacid and antiulcer (133 (34.6%)) as shown in Table 2. The standard treatment guideline, formulary manual, and essential list for Ethiopian were found at the pharmacy department head office. The former hospital Drug Therapeutic Committee (DTC) was the one that developed the essential list of medicine for the hospital; it was revised 3 years back (2014).

**Figure 1:**
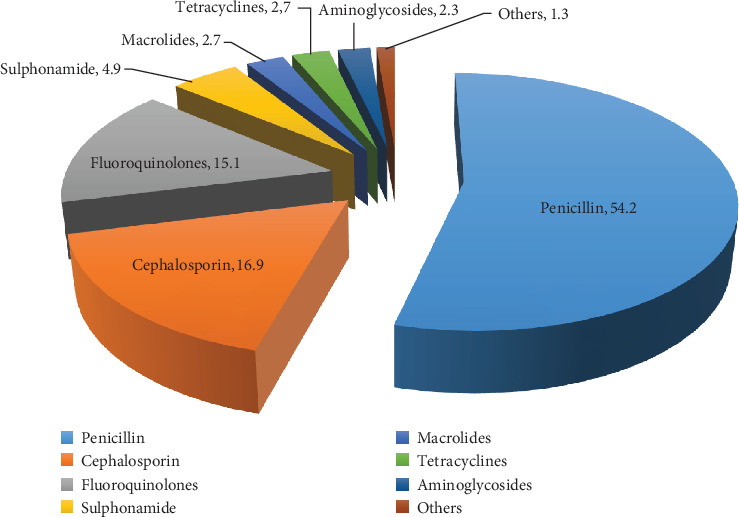
Frequency of prescribing antibacterial by group in MGH from January 1 to December 31, 2016.

Among 225 (58.6%) prescriptions which contained antibiotics, the most prescribed groups of antibiotics were penicillin 122 (54.2%), cephalosporin 38 (16.9), and fluoroquinolones 34 (15.1%)(Figure 1).

## 4. Discussion

This study has provided a better understanding of the prescribing practices in MGH and has shown areas that need intervention by using the WHO prescribing indicators. These indicators measure the performance of a health care provider related to the appropriate use of drugs. In the present study, the average number of drug per prescription, which measures polypharmacy, was 1.96. The value was higher than the WHO recommended value of 1.6-1.8. Similarly, the value was higher than the studies conducted in North Ethiopia Felege Hiwot Referral Hospital (1.83) [10], Northeast Ethiopia Boru Meda Hospital (1.88) [11], and Hawassa University Teaching and Referral Hospital (1.9) [4]. And the result was smaller than the study conducted in West Ethiopia four public hospitals (Ambo, Gedo, Nekemet, and Gimbi) which was 2.1 [5], Ayder Referral Hospital (2.61) [6], and Northwest Ethiopia Debremarkos Hospital (2.4) [12]. The present study also showed that the average number of drugs per encounter was less than a study conducted in five national regional states (Tigray, Amhara, Oromia, SNNPR, and Benishangul-Gummuz) and Addis Ababa in a total of 140 health facilities revealed result (1.99) [13]. Higher results were reported in studies abroad; in Kenya, the study showed that the average number of drugs per prescription was 2.7 [14], Nigeria 3.04 [15], India 3.11 [16], and Ghana 4.8 [17]. Another study conducted in Bahrain showed 3.3 drugs per encounter [18]. In Pakistan, the study presented 3.4 drugs per prescription [19]. The research conducted in different health facilities of Nepal, United Arab Emirates (UAE), Uzbekistan, and Jordan displayed 2.29, 2.49, and 2.9, 2.93, respectively. Even though the studies conducted in these three countries are lower than Nigeria, India, Bahrain, Pakistan, and Ghana, the results indicated that there were prescribing practices higher than the WHO recommendation [20–23].

The average number of drugs per prescription in MGH was beyond from the WHO recommendation. The likely negative effects of prescribing many drugs per prescription are increased incidences of side effects, drug-drug interactions, confusion where aged patients are involved, noncompliance by patients to the drug regimen, and increase cost of pharmacotherapeutic as a result of the large number of drugs to be taken at a time and for prolonged periods in most cases [10].

Low generic prescribing is seen in this study; from 751 drugs, only 679 (90.4%) drugs were prescribed by their generic name. This finding is smaller than that of the WHO recommendation (100%) [9]. Studies conducted in different health facilities revealed that there were better generic prescribing practice, like Hawassa University Teaching and Referral Hospital 98.7% [4] North Ethiopia Felege Hiwot Referral Hospital 97.4% [10], and Ayder Referral Hospital 93.3% [6]. Similar studies were conducted in other governmental hospitals which displayed that generic prescribing was seen lesser than MGH, like Northwest Ethiopia Debremarkos Hospital 77.7% [12], Four west Ethiopia public Hospitals (Ambo, Gedo, Nekemet, and Gimbi) 79.2 [5], and Northeast Ethiopia Boru Meda Hospital 80.02% [9]. Studies conducted abroad show much smaller than generic prescribing from the result found in MGH. 45.5% and 42.7% in Kenya and Nigeria, respectively [14, 15], but in India generic prescribing was 96.88% [16]. Another study conducted in Ghana show that generic prescribing was low. Only 65% of prescriptions contained the generic name of the drugs [17]. In addition to this, the study conducted in Bahrain revealed very low generic prescribing, 10.2% [18]. Studies conducted in Pakistan, Nepal, Jordan, and Uzbekistan publicized that 71.6%, 59.02%, 57.6%, and 38%, respectively [19, 20, 22, 23]. A study done in UAE showed that the average number of drugs prescribed with their generic name was 100% [21] which is in line with WHO recommendation.

Low generic prescribing could add confusion of patients who are already faced with the burden of polypharmacy. This could lead to duplication errors where patients may unknowingly take the generic and brand products of the same drug simultaneously. Generic prescribing is an indicator of prescribing quality [10].

Rates of antimicrobial resistance are increasing in health facilities and the community. The prevalence of antimicrobial resistance in any population is related to the proportion of the population that receives antimicrobial and total antimicrobial exposure. Increased antimicrobial use leads to more resistance [24]. In the present study, antibiotics were prescribed in 225 (58.6%) of the total prescriptions which is almost double the upper limit of the WHO recommendation (20-26.8%) [9]. Many studies' results showed that higher antibiotic prescribing practice is common in Ethiopia. It was 71.36% in Debremarkos Referral Hospital [12], 58.1% in Hawassa University Teaching and Referral Hospital [4], 58% in five national regional states (Tigray, Amhara, Oromia, SNNPR, and Benishangul-Gummuz) and Addis Ababa, in a total of 140 health facilities [13], and 54.7% in four west Ethiopia public Hospitals (Ambo, Gedo, Nekemet, and Gimbi) [5]. It also revealed 38%, 34.57%, 32%, and 24.37% in Felege Hiwot Hospital, Boru Meda Hospital, and Ayder Referral Hospital, respectively [6, 10]. Similarly, a study conducted in Kenya showed that majority of prescriptions (74%) contained antibiotics [14]. In Nigeria, the percentage of prescriptions involving antibiotics averaged 34.4% [15]. A study conducted in Ghana shows that 60% of prescriptions contained antibiotics. This is comparable to the current study [17]. In other studies conducted in Bahrain, Pakistan, Nepal, and Uzbekistan it was declared that 45.8%, 48.9%, 57%, 60.9%, and 57%, respectively, of the prescriptions were with antibiotics [18–20, 22]. On the contrary, good results were found in studies done in Dessie Referal Hospital (Ethiopia), Indian Hospital, in health facilities of Jordan and UAE with values of 24.37%, 22.19%, 17.7% and 9.8%, respectively [11, 16, 21, 23], which are even below the WHO recommendation.

Unsafe and overuse of injections play an important role in the transmission of very serious blood-borne infections and leads to disability and death. In the present study, injections were prescribed in 162 (42.2%) of the total prescriptions. The finding was much higher than the WHO recommendation (13.4-24.1) [9]. A study done in Debremarkos Referral Hospital showed that 48.36% prescriptions were injectable [12], 38.1% was revealed in Hawassa University Teaching and Referral Hospital [4], and 26.9% was shown in five national regional states (Tigray, Amhara, Oromia, SNNPR, and Benishangul-Gummuz) and Addis Ababa in a total of 140 health facilities [13]. Lower percentage of encounters containing injections was seen in Boru Meda Hospital and Felege Hiwot, 6.06% and 10.8%, respectively [1, 11]. Similarly, studies conducted in Nigeria and Kenya had shown values of 4% and 13.2%, respectively [14, 15] that are in line with the recommendation [9] and India 22.19% [16]. A study conducted in Ghana stated 80% of encounters had injections. The result showed extremely high injection prescription per encounter and this study almost doubled the results found in the current study and more far to the standard limit of WHO [17]. Another studies conducted in Pakistan, India, Bahrain, Jordan, UAE, and Nepal disclosed results of 27.1%, 22.19%, 9.3%, 8.1%, 3.14%, and 3%, respectively. All results shown in these studies were lower than the standard lower limit of WHO except Pakistan [16, 18–21, 23].

86.3% of drugs were listed in MGH's essential drug list. This value is too far to that of the recommended by WHO (100%) and also lower than other reports from different health facilities in Ethiopia. From which, Felege Hiwot Referral hospital and Ayder Referral Hospital adhered 100% with EML [6, 10] and others like Debremarkos Referral Hospital and Hawassa University Teaching and Referral Hospital were 98.24% and 96.6%, respectively [4, 12] which are close to WHO recommendations (100%). On the other hand, four west Ethiopia public hospitals (Ambo, Gedo, Nekemet, and Gimbi), and Boru Meda Hospital encountered 83% and 85.26%, respectively [5, 11]. Our study result is comparable to studies done in Nepal, 85.19% [20], but lower than from Pakistan, 93.4% [19], Jordan, 99.8% [23], and UAE, 100% [21]. Even if the results from these studies were better than the current study, except UAE, no one had obeyed the WHO recommendation.

## 5. Conclusion

In general, average number of drugs per encounter, generic prescribing, and the use of EML/formulary of the hospital to prescribe drugs reviewed in this study were totally out of the recommended values and hence inappropriate. The study also revealed overprescribing of both antibiotics and injections. To ensure the rational use of drugs in Mekelle General Hospital, a number of recommendations can be suggested based on the findings. First, there should be a strong and fully functionalized drug and therapeutic committee in the hospital. Second, the existing hospital list of drugs should be revised. Third, there should be continuous sensitization trainings for prescribers and pharmacists (dispensers) on the rational use of medicines. Fourth, clinical pharmacists who are working in the hospital should take responsibility and actively participate in drug prescribing and dispensing practice especially in the outpatient department. Fifth, a copy of the treatment guideline and hospital essential list of drugs should be availed in all outpatient prescribing and dispensing units.

## Figures and Tables

**Table 1:** Drug prescribing indicators in MGH from January 1, 2016 to December 31, 2016 (*n* = 384).

Prescribing indicators	Number	Average/percentage	Ideal WHO value [9]
Average number of drugs per encounter	751	1.96	1.6-1.8
Percentage of drugs prescribed by generic	679	90.4%	100%
Percentage of encounter with antibiotics	225	58.6%	20.0-26.8%
Percentage of encounter with injections	162	42.2%	13.4-24.1%
Percentage for drugs from drug list of MGH	648	86.3%	100%

**Table 2:** The most common prescribed drug classes in MGH, 2016.

Prescribing indicators	Value (%)
Encounter with antibiotic	225 (58.6%)
Encounter with analgesics and antipyretics	164 (42.7)
Encounters with antacid and antiulcer	133 (34.6)
Encounter with narcotic and psychotropic	37 (9.6)
Encounter with vitamins and minerals	34 (8.85)
Encounters with cardiovascular including antihypertensive	27 (7.03)
Encounter with anthelmintic	20 (5.2)
Encounter with steroid and hormonal preparations	17 (4.4)
Encounter with antitussive (cough suppressant)	16 (4.2)
Encounter with antihistamine	15 (3.9)

## Data Availability

Data will be available on request. The corresponding author can be contacted (Zewdu Yilma: ziedo21@gmail.com).

## Abbreviations

DTC: Drug therapeutic committee
EML: Essential medicine list
INRUD: International network for rational use of drugs
IPLS: Integrated Pharmaceutical Logistics System
MGGH: Mekelle General Hospital
NGO: Nongovernmental organization
OPD: Outpatient department
SNNPR: Southern Nations, Nationalities, and Peoples' Region
WHO: World Health Organization.
